# Inflammatory Biomarkers for Assessing In-Hospital Mortality Risk in Severe COVID-19—A Retrospective Study

**DOI:** 10.3390/jpm14050503

**Published:** 2024-05-10

**Authors:** Erika Bimbo-Szuhai, Mihai Octavian Botea, Dana Diana Romanescu, Corina Beiusanu, Gabriela Maria Gavrilas, Georgiana Maria Popa, Dania Antal, Mihaela Gabriela Bontea, Liliana Sachelarie, Iulia Codruta Macovei

**Affiliations:** 1 Department of Morphological Disciplines, Faculty of Medicine and Pharmacy, University of Oradea, 410073 Oradea, Romania; bszera@gmail.com (E.B.-S.); gabriela_gavrilas78@yahoo.com (G.M.G.); bontea.mihaela@yahoo.ro (M.G.B.); 2 Pelican Hospital, 410450 Oradea, Romania; drmob78@yahoo.com (M.O.B.); danamed2000@yahoo.com (D.D.R.); pascalau.georgiana17@yahoo.com (G.M.P.); dania.tinca@yahoo.com (D.A.); icmek69@gmail.com (I.C.M.); 3 Department of Surgery, Faculty of Medicine and Pharmacy, University of Oradea, 410073 Oradea, Romania; 4 Department of Medical Disciplines, Faculty of Medicine and Pharmacy, University of Oradea, 410073 Oradea, Romania; 5 Department of Preclinical Disciplines Apollonia, Faculty of Medicine, University from Iasi, 700511 Iași, Romania

**Keywords:** COVID-19 infection, G/L ratio, mortality predictors in ICU

## Abstract

(1) Background: Our study aims to investigate the utility of inflammatory factors as prognostic indicators for disease severity and mortality in COVID-19 patients admitted to the Intensive Care Unit (ICU) Department of Pelican Clinical Hospital Oradea Romania. While elevated white blood cell (WBC) levels are associated with COVID-19 severity and mortality, they may not effectively predict the risk of death; (2) Methods: In our ICU department, we conducted assessments on the 10th and 14th days of COVID-19 patients’ hospitalization, measuring the following markers: C-reactive protein (CRP) levels, procalcitonin (PCT) levels, granulocytes/lymphocytes (G/L) ratios, ferritin levels, age, and obesity status. We included a total of 209 eligible COVID-19 patients in the final analysis. Our goal was to identify biomarkers that could quickly identify high-risk patients with a potential for disease progression and mortality; (3) Results: Our study (a retrospective, single-center observational cohort study) demonstrated statistically significant differences in predicting mortality and disease severity based on G/L ratio (*p* < 0.0001), PCT (*p* < 0.0002), CRP (*p* < 0.0001), ferritin (*p* < 0.0001), age (*p* < 0.0001), and obesity (*p* < 0.0001); (4) Conclusions: Having a G/L ratio exceeding 20 units, along with elevated levels of PCR, PCT, and ferritin in older and obese patients on the 3rd day of ICU admission, represents significant risk factors for in-hospital mortality in severe COVID-19 patients.

## 1. Introduction

In late 2019, China reported the first cases of pneumonia with an unknown cause. By the end of January 2020, the World Health Organization declared this outbreak a Public Health Emergency of International Concern [1].

A systematic analysis for the Global Burden of Disease Study published in 2021, demonstrate that COVID-19 replaced stroke, and in this way, stroke became the third leading cause of death [2]. COVID-19 presents with nonspecific symptoms and clinical manifestations can range from asymptomatic cases to severe forms of pneumonia and acute respiratory distress syndrome (ARDS). The majority of infected individuals experience mild symptoms, including fever, cough, myalgia, fatigue, and shortness of breath. However, some cases progress to severe respiratory symptoms, such as pulmonary edema, ARDS, and multi-system organ failure (MSOF), ultimately resulting in death [3]. Given the potential for severe disease development, it is crucial to quickly assess patients’ progression.

Continuous monitoring of biological markers is essential to evaluate disease progression. Biomarkers play a pivotal role in clinical practice as they can indicate the evolution of various pathological conditions and serve as indicators for patient monitoring, influencing treatment decisions.

A primary concern in clinical practice is the rapid transition from mild to severe disease. It is recommended to identify these possible progressions early and provide timely therapeutic interventions. Various methods for classifying and stratifying the severity and mortality risk in COVID-19 disease have been developed. Changes in biomarker levels can indicate the progression of patients toward severe cases, making them valuable tools in clinical practice to guide treatment decisions and determine the need for admission to the Intensive Care Unit (ICU).

Numerous studies have highlighted changes in biomarkers associated with the progression of COVID-19, particularly in severe cases. These biomarkers include white blood cell count (WBC), C-reactive protein (CRP), D-dimer, interleukin-6 (IL-6), lactate dehydrogenase (LDH), urea, and creatinine, all of which tend to be elevated in COVID-19 disease [4,5].

It has been established that inflammation plays a pivotal role in the progression towards severe forms of COVID-19 disease. The severe inflammatory response observed in COVID-19 is a consequence of an inappropriate immune response, often stemming from a compromised adaptive immune function. Patients with severe disease require a more comprehensive evaluation to identify the optimal moment for potential reconsideration of treatment in the Intensive Care Unit (ICU), if necessary. In this context, early identification of risk factors is essential. The identification of hyper-inflammation through the use of biological markers is crucial. This is particularly important due to the diverse clinical presentations and variable disease progression observed in COVID-19 patients. The ability to detect hyper-inflammation helps healthcare providers make informed decisions about treatment adjustments based on the patient’s clinical progress [6].

Several studies have recognized the neutrophil-to-lymphocyte ratio (NLR) as a valuable tool for assessing inflammatory status due to its cost-effectiveness and ease of access. NLR has been employed as a potential biomarker in evaluating various conditions associated with a proinflammatory state, including chronic obstructive pulmonary disease (COPD), pancreatitis, colorectal cancer, and cardiovascular disease [3].

NLR is a biomarker that tends to be elevated in widespread inflammatory diseases and serves as a reflection of disease severity and progression [4,7,8,9,10].

In the current healthcare landscape, there exists an urgent demand for the development of novel mortality predictors. These predictors are essential for healthcare professionals to accurately assess the risk of in-hospital death in patients afflicted with severe forms of coronavirus infection. We advocate for the implementation of a comprehensive set of routine laboratory parameters to be systematically measured upon admission for individuals with severe COVID-19 who necessitate admission to the Intensive Care Unit (ICU). This panel of parameters encompasses the granulocyte-to-lymphocyte ratio (G/L ratio), C-reactive protein (CRP), ferritin, procalcitonin, age, and obesity status.

The objective of these measurements is to effectively forecast the progression of severe COVID-19 manifestations and the associated risk of mortality in these patients. Ensuring the continual monitoring of patients, both clinically and through laboratory assessments, is of paramount importance. Such vigilance aids in the early estimation of mortality risk at the time of hospital admission for individuals with COVID-19, particularly those requiring hospitalization. The pursuit of reliable, sensitive, and specific biomarkers plays a pivotal role in this endeavor, enhancing our capacity to provide timely and targeted medical care.

A study conducted by Elliott et al. [11] has confirmed the relationship between adipose tissue area, body mass index (BMI), airway wall thickness, and inflammation. Another meta-analysis conducted by Foldi et al. [12] demonstrated that obese patients with COVID-19 disease are more likely to require invasive mechanical ventilation (IMV), underscoring obesity as a significant risk factor in disease progression.

Patients aged 60 years and older tend to experience more severe and prolonged clinical manifestations compared to those under 60 years of age [13,14]. The prognosis is less favorable for patients above 60 years of age, prompting healthcare professionals to exercise heightened vigilance in managing this age group.

This study aims to assess the role of various biomarkers in predicting the progression of COVID-19 disease and to provide clinicians with a tool for forecasting prognosis and mortality in patients with severe COVID-19 disease admitted to the Intensive Care Unit (ICU). There is an imperative need for an economical, rapid, and reliable marker for predicting in-hospital mortality in severe COVID-19 cases. The use of the granulocyte-to-lymphocyte (G/L) ratio as a means to estimate the risk of in-hospital death in critically ill COVID-19 patients may facilitate the early identification of individuals requiring more aggressive therapeutic interventions, ultimately reducing mortality rates. Our investigation aims to determine if G/L ratios can serve as a valuable prognostic biomarker for disease severity and mortality in COVID-19 patients.

## 2. Materials and Methods

### 2.1. Study Subjects

Our objective was to identify biomarkers that could expedite the assessment of patients at high risk of experiencing worsening disease progression and death.

After conducting an extensive review of the current literature and considering the routine biological markers available in our ICU department, we proposed to monitor the following parameters on the 10th and 14th days of COVID-19 patients’ hospitalization: granulocyte-to-lymphocyte ratios (G/L), C-reactive protein (CRP), procalcitonin, and ferritin levels.

Our clinical study took the form of a retrospective, single-center observational cohort study. It included consecutive patients, all of whom had been diagnosed with COVID-19 and were admitted to the ICU Department of Pelican Clinical Hospital Oradea, Romania between January 2021 and December 2021. In our country, after a positive PCR-test, all patients were admitted to hospitals for treatment and surveillance.

The entire study protocol was analyzed and approved by the Hospital’s Ethics Committee nr.202/15.12.2020 in compliance with the Declaration of Helsinki and its amendments.

Due to the retrospective nature of our study and the circumstances of the patients involved, the requirement for written informed consent was waived. This decision was made considering the unique design of this study and the condition of the patients, where obtaining written consent may not have been feasible or appropriate. 

This study aimed to analyze existing data for the purpose of improving medical understanding and patient care, while ensuring patient confidentiality and ethical considerations.

### 2.2. Study Participants and Inclusion and Exclusion Criteria

A total of 220 patients were included in this study. These patients met the criteria for enrollment, which required them to have tested positive for SARS-CoV-2 infection through at least one Reverse Transcription Polymerase Chain Reaction (RT-PCR) test conducted on nasopharyngeal and/or oropharyngeal swabs. Additionally, these patients needed to exhibit evidence of lung damage as determined by thoracic computer tomography (CT) scans, which were independently interpreted by a senior radiologist. These criteria ensured that this study focused on individuals with confirmed COVID-19 and pulmonary involvement.

Exclusion criteria were age under 18 years, pregnant women, patients previously diagnosed with hematological disorders, cancer, rheumatoid arthritis, and severe comorbidities before COVID-19 disease, because that may have an essential impact on laboratory parameters. Another exclusion criterion is represented by patients who died on the first three days after admission. A total of nine patients met these exclusion criteria. No patients were excluded based on their sex, place of origin, or ethnicity.

**Figure 1:**
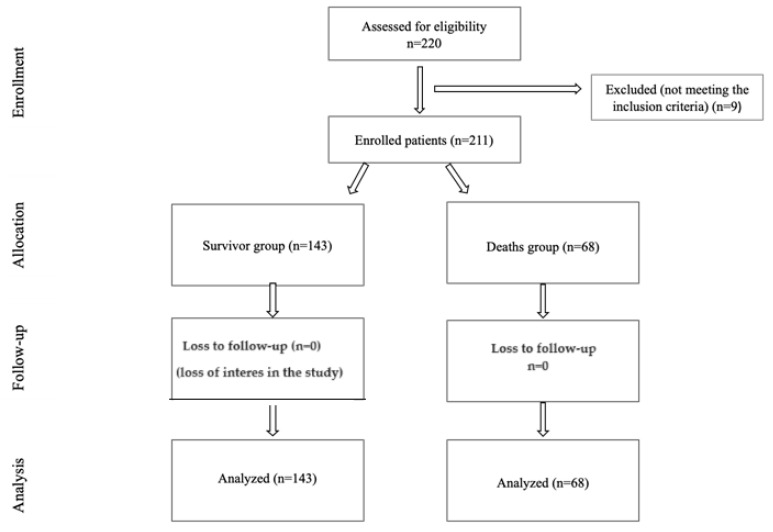
Flow consort.

Considering these inclusion and exclusion criteria, a final analysis was conducted on a total of 211 eligible COVID-19 patients who met this study’s criteria (refer to Figure 1).

### 2.3. Data Collection

The data used in this study, which included demographic information, laboratory results, and patient outcomes, were retrieved from electronic medical records and meticulously reviewed by clinical physicians.

Blood samples were collected from patients after fasting, and collection was conducted through a central catheter. Venous blood was drawn and collected in specialized vacutainers with anticoagulants for whole blood counts and leukocyte formulae (used to calculate the G/L ratio). For biochemical determinations such as ferritin, procalcitonin, and C-reactive protein (CRP) levels, venous blood was collected in vacutainers without anticoagulants.

The laboratory analyses were conducted using the following methods:

✓ An automatic analyzer that employs flow cytometry with fluorescence, utilizing a semiconductor LASER and hydrodynamic focusing for blood count and leukocyte formula determination.
✓ A latex-enhanced immunoturbidimetric method for assessing CRP and procalcitonin levels.

These rigorous procedures were followed to ensure accurate and reliable data collection and analysis for this study.

### 2.4. Statistical Analysis 

For the management and analysis of the collected data, the medical statistics program MedCalc^®^ version 12.5.0.0 (MedCalc^®^ Software, Mariakerke, Belgium) was employed. This software was used to store the information entered into the study database and to conduct various statistical analyses.

The outcomes of the statistical tests are reported using the probability of the “null” hypothesis (*p*). A *p*-value below 0.05 is indicative of a statistically significant difference between the groups being studied. Additionally, specific results will be visually represented in graphical format, utilizing the same statistical program for this purpose.

MedCalc^®^ played a crucial role in processing and interpreting the data, allowing for the identification of significant findings and trends within this study’s parameters.

The choice of statistical tests in our study depended on the nature of the variables being analyzed. Parametric tests, suitable for variables with a normal distribution, included the Student’s *t*-test for independent groups. Non-parametric tests, appropriate for variables with asymmetric distributions, encompassed the Mann–Whitney test and the Kaplan–Meier test. For categorical variables, we presented their descriptions using both absolute values and percentages (in brackets). We employed the chi-square test with Yates’ correction to examine and analyze categorical data, assessing associations and differences as necessary.

These diverse statistical methods allowed us to comprehensively analyze various types of data. 

## 3. Results

### Characteristics of the Population

Following the application of admission and discharge criteria to all patients admitted for SARS-CoV-2 infection in the ICU Department of Pelican Hospital during the period of January 2021 to December 2021, a total of 211 patients were identified and included in this retrospective study. Among these patients, there were 68 cases (representing an in-hospital mortality rate of 32.2%), who died during hospitalization, Table 1.

Additionally, the assessment of biochemical parameters conducted upon admission played a critical role in evaluating the clinical status of the patients and predicting the potential for a severe clinical course. 

These results are systematically compared and presented in Table 2.

All the measured inflammatory parameters displayed significant disparities between the deceased and the survivors. Specifically, an elevated count of leukocytes and neutrophils, coupled with a reduced number of lymphocytes, was linked to a heightened risk of in-hospital mortality. Furthermore, an increased granulocyte-to-lymphocyte ratio was more frequently observed in patients who ultimately experienced fatal outcomes. Additionally, elevated plasma levels of C-reactive protein, ferritin, and procalcitonin were also linked to a greater risk of in-hospital mortality.

The dynamic changes in these values, particularly when reevaluated on the 14th day of hospitalization during ICU admission, are comprehensively presented in Table 3. These trends offer crucial insights into the evolving inflammatory responses and their correlation with patient outcomes.

There are statistically significant differences between the survivor and deceased groups persisting on the 4th day of ICU admission (14th day of hospitalization) (*p* < 0.0001), Table 3.

It is notable that no significant differences were observed between the groups of patients in terms of treatment regimens or the duration of hospitalization, Table 4.

The analysis of risk factors for in-hospital death following SARS-CoV-2 infection was conducted by constructing a logistic regression model with the stepwise entry of all variables that had shown significant differences in the previous statistical studies. This analysis has identified the following variables as independent risk factors with a *p*-value of less than 0.0001 (refer to Table 5). These findings contribute to our understanding of the factors associated with in-hospital mortality among patients with SARS-CoV-2 infection and can inform clinical decision-making and patient management.

Obesity and procalcitonin levels exceeding 10 ng/mL, both at the time of admission and on the 14th day of hospitalization, are risk factors for ICU mortality. 

Procalcitonin levels of 10 ng/mL or higher are rarely encountered, and none of the patients exhibiting such elevated levels survived throughout their hospitalization. Within this study cohort, obesity emerges as an independent risk factor.

This study indicates that patients who are over 60 years old with obesity and with a granulocyte-to-lymphocyte ratio exceeding 10 on the 14th day after admission to the hospital are at a higher risk of in-hospital mortality following SARS-CoV-2 infection. 

**Figure 2:**
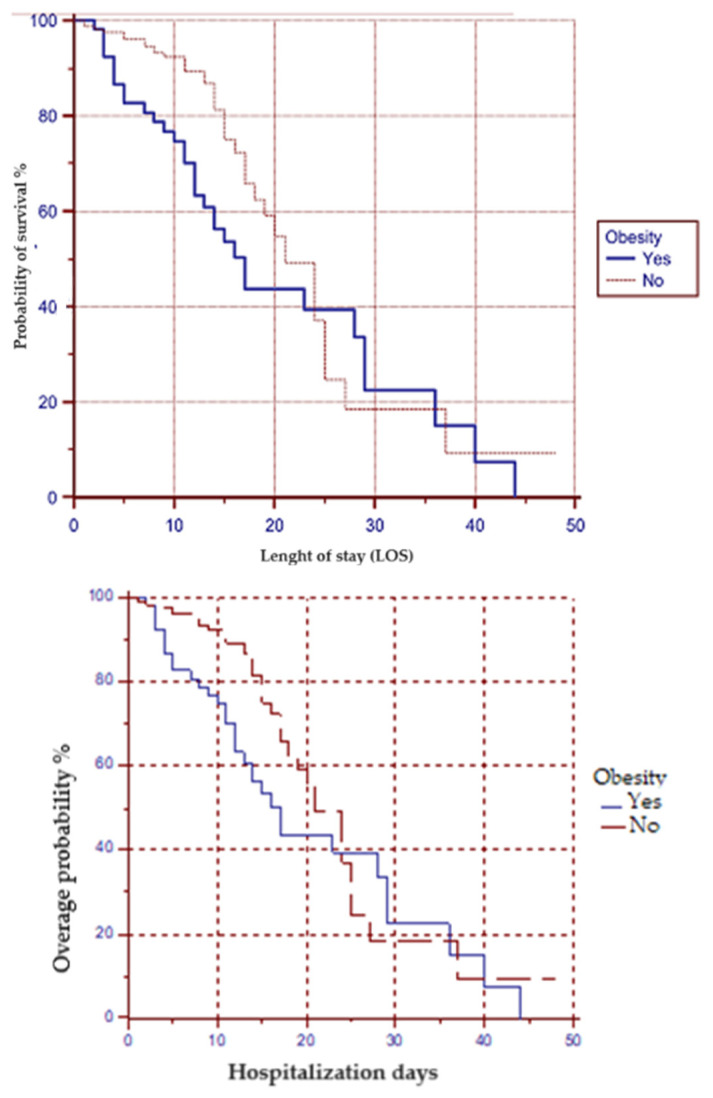
In-hospital survival curve for patients with and without obesity hospitalized for SARS-CoV2 infection.

The Kaplan–Meier survival curve, comparing patients with and without obesity indicates notable differences in patient outcomes (*p* = 0.0315, see Figure 2). These findings emphasize the critical impact of obesity as a risk factor for mortality in patients admitted to the ICU due to SARS-CoV-2 infection.

Among the continuous variables, two others that have emerged as independent risk factors for in-hospital death are older age (over 60 years) and an increased granulocyte-to-lymphocyte ratio on the 14th day of hospitalization (4th day of ICU admission). The area under the receiver operator curve (AUC) analysis for these two variables yielded the following results:

- For age, the AUC is 0.7157 with a *p*-value of less than 0.0001. The recommended threshold is over 60 years, with a Youden J index of 0.3579. This threshold provides a sensitivity of 88.24% and specificity of 47.55% for predicting in-hospital death.
- For the granulocyte-to-lymphocyte ratio on the 14th day of hospitalization (4th day of ICU admission), the AUC is 0.7789 with a *p*-value of less than 0.0001. The recommended threshold is over 10, with a Youden J index of 0.4528. This threshold offers a sensitivity of 69.64% and specificity of 74.19% for predicting in-hospital death.

This study indicates that patients who are over 60 years old, obese, and those with a granulocyte-to-lymphocyte ratio exceeding 10 on the 14th day of hospitalization are at a notably higher risk of in-hospital mortality following SARS-CoV-2 infection. 

These identified risk factors can serve as valuable indicators for healthcare professionals in assessing and managing the prognosis of COVID-19 patients, particularly those admitted to the ICU.

## 4. Discussion

In this study we analyzed a total of 211 eligible patients with COVID-19. Evaluating the G/L ratio as a prognostic value for disease severity and mortality in infected patients, we found a moderate negative correlation with outcome. Finally, it can be used as a rapid and reliable economic marker for in-hospital mortality.

An elevated G/L ratio exceeding 20 G/L is a risk factor for in-hospital mortality among patients with severe COVID-19 disease. The G/L ratio serves as a reliable and consistent biomarker for predicting both disease severity and probability of mortality. Notably, surviving patients generally had a lower G/L ratio. Previous studies have evaluated numerous laboratory biomarkers to predict poor prognosis of COVID-19, which may be indicative of inflammatory conditions and signs of organ dysfunction or damage [15,16]. Our study showed a statistically significant difference in the prediction of mortality and severity using G/L ratio, PCT ferritin, CRP level, age, and obesity.

Our data showed that G/L ratio leucocytes, ferritin and CRP level, age, and obesity (*p* < 0.0001) have a stronger relevance to outcome compared to PCT (*p* < 0.0002). In other words, the use of G/L ratio may assist clinicians in quickly identifying patients at higher risk of mortality with the aim of giving them priority in ICU admission.

The severity and mortality of COVID-19 have been closely associated with the extensive infiltration of neutrophils in the lungs and elevated neutrophil counts in peripheral blood. The degree of neutrophilia often reflects the intensity of inflammatory responses [17]. Elevated levels of inflammatory biomarkers, observed in both severe and critical COVID-19 cases, indicate that COVID-19 triggers potent inflammatory responses which may be linked to unfavorable clinical outcomes [18]. Regarding the role of obesity [19,20,21], this condition enhances the inflammation or inflammatory response in COVID-19 patients. 

Comparative studies between COVID-19 and influenza patients have shown higher rates of bacterial infections in COVID-19 patients. Interestingly, these bacterial infections were more common in fatal cases [22,23,24]. Critically ill COVID-19 patients had the highest percentage of bacterial coinfections compared to moderately or severely ill patients [25,26]. Dysregulated virus-induced immune responses are thought to contribute to bacterial colonization, and neutrophils play a critical role in controlling bacterial infections [27,28].

The identification of high mortality markers in patients can aid in early recognition, prompting more aggressive therapeutic management. In addition to the criteria used for stratifying COVID-19 disease severity, various clinical scoring systems have been proposed, including the widely used APACHE II scoring; COVID-19 Critical Illness Prediction Tool (COVID-GRAM); SOFA score; and Comorbidity, Age, Lymphocyte Count, Lactate Dehydrogenase score (CALL score) [29].

Several research groups, primarily from China, have suggested that serum levels of ferritin, CRP, and procalcitonin are reliable predictors of mortality in patients with severe COVID-19 [30,31,32]. The G/L ratio was found to have a moderate negative correlation with patient outcomes, indicating that as the G/L ratio decreases, patient outcomes tend to improve. Age and procalcitonin also exhibited a moderate negative correlation with outcomes. The correlation between outcomes and the number of days spent in the Intensive Care Unit (ICU) was found to be weak. These insights into the relationship between various biomarkers and patient outcomes provide valuable information for healthcare professionals in assessing and managing patients with severe COVID-19 disease.

Indeed, some clinical scoring systems like APACHE II and COVID-GRAM involve a relatively large number of variables, including those that rely on advanced laboratory tests such as lactate dehydrogenase (LDH), serum electrolytes, and arterial pH. Additionally, biomarkers like interleukin-6, D-dimer levels, C-reactive protein (CRP), and soluble urokinase plasminogen activator receptor (suPAR) can be expensive and inaccessible, making them challenging to implement in healthcare settings, especially in Low and Low–Middle-Income Countries (LICs/LMICs) with limited resources [33,34,35].

Hence, the appeal of simpler tools like the neutrophil-to-lymphocyte ratio (NLR) lies in their quick turnaround and cost-effectiveness. Our study highlights the significance of age and obesity in conjunction with a G/L ratio above 10 on the 3rd day after admission as risk factors for in-hospital mortality following SARS-CoV-2 infection.

Age has consistently emerged as one of the most significant risk factors for various COVID-19 outcomes [36]. Patients with one or more pre-existing medical conditions also face a higher risk of severe outcomes [17,18,22]. Numerous studies have established the association between pre-existing comorbidities and increased mortality risk [37,38]. Among these comorbidities, diabetes and obesity have prominently stood out as dominant risk factors for mortality in COVID-19 patients [39,40]. Additionally, chronic obstructive pulmonary disease (COPD) has been noted as a pre-existing condition in fatal cases of COVID-19 patients [30]. However, in our study, only a limited number of eight patients with COPD were admitted to the ICU for severe progression, resulting in a non-significant difference between the two groups (*p* = 0.9519).

The levels of biomarkers have the potential to change in accordance with the severity of COVID-19 disease, making them valuable tools in clinical practice for guiding treatment decisions, optimizing ICU admissions, and ultimately improving patient prognosis while minimizing mortality rates [40]. The finding that a G/L ratio exceeding 20 on the 3rd day after ICU admission serves as a risk factor for in-hospital mortality in patients with severe COVID-19 disease is significant. Additionally, the consistency of the G/L ratio, CRP, procalcitonin (PCT), ferritin, age, and obesity as biomarkers for predicting both disease severity and mortality reinforces their importance in clinical practice.

Our analysis suggests that inflammatory markers such as the G/L ratio could potentially be used as predictors of in-hospital mortality for critical COVID-19 patients. An important point regarding case evolution in our country at the beginning of the pandemic is that it was a protocol to admit in hospital, for at least 14 days, newly diagnosed cases having cardiovascular disease and obesity as comorbid conditions. The only demographic parameter that presented significant differences between survivors and non-survivors in our research was the patients’ age.

Our study presents its limitations. It is a retrospective study with a relatively small number of patients (211) from a single center during a certain period of the pandemic, with no data available regarding vaccination statuses of the patients.

## 5. Conclusions

Our study has demonstrated that the granulocyte-to-lymphocyte (G/L) ratio, CRP, ferritin, procalcitonin, age, and obesity can collectively serve as economic, rapid, and reliable markers for predicting in-hospital mortality. The described biomarker is cheap, easy, and simple to calculate. Patients enrolled in our study, diagnosed with COVID-19, were admitted to the ICU Department of Pelican Clinical Hospital Oradea Romania.

These markers hold significant potential for aiding clinicians in assessing the risk of mortality in COVID-19 patients, allowing for timely and informed decision-making in clinical practice.

These economic and reliable markers for in-hospital mortality in severe COVID-19 disease have the potential to play a crucial role in assisting clinicians. By quickly identifying patients at a higher risk of mortality, these markers can help prioritize their admission to Intensive Care Units, ultimately contributing to more effective patient management and care.

## Figures and Tables

**Table 1:** Demographic data and pathological history for the two study groups.

	Survivors (n = 143)	Deaths (n = 68)	Statistical Significance (*p*)
Gender (M/F)	62/81	32/36	0.7207 *
Age (years)—median (IQR)	62 (48–71)	72 (64–77)	<0.0001 **
Environment of origin (U/R)	113/30	44/24	0.0396 *
History of COPD—no. of patients (%)	5 (3.5%)	3 (4.4%)	0.9519 *
History de DM—no. of patients (%)	29 (20.3%)	24 (35.3%)	0.0292 *
History of depression—no. of patients (%)	3 (2.1%)	2 (2.9%)	0.9141 *
Obesity—no. of patients (%)	21 (14.7%)	32 (47.1%)	<0.0001 *

M = male. F = female. IQR = interquartile range. U = urban. R = rural. COPD = chronic obstructive pulmonary disease. DM = diabetes mellitus; *—chi-square test with Yates’ correction; **—Mann–Whitney test. In the context of medical data analysis, it is important to note that among the demographic variables examined, age and patients’ areas of origin exhibited statistically significant differences. Specifically, it was observed that the patients who unfortunately passed away in the hospital tended to be older on average and were predominantly from rural areas. Regarding medical history, it is noteworthy that there was a significantly higher prevalence of diabetes and obesity among patients who did not survive their hospitalization.

**Table 2:** Biochemical parameters in ICU for both study groups.

Biochemical Parameters at the Time of Admission to ICU	Survivors (n = 143)	Deaths (n = 68)	Statistical Significance (*p*)
Leukocytes (×10^3^/mm^3^)—median (IQR)	6.58 (4.7–8.9)	10.74 (6.3–15.3)	<0.0001 *
Neutrophiles (×10^3^/mm^3^)—median (IQR)	4.84 (3.0–7.3)	9.29 (5.9–13.6)	<0.0001 *
Lymphocytes (×10^3^/mm^3^)—median (IQR)	0.91 (0.6–1.4)	0.61 (0.4–0.9)	<0.0001 *
Granulocytes/Lymphocytes ratio—median (IQR)	4.77 (2.6–9.4)	14.74 (7.0–28.3)	<0.0001 *
Procalcitonin (ng/mL):			
<0.5	129 (90.2%)	46 (67.6%)	0.0002 **
0.5–2	13 (9.1%)	16 (23.5%)	
2–10	1 (0.7%)	4 (5.9%)	
>10	0 (0.0%)	2 (2.9%)	
CRP (mg/L)—median (IQR)	44.35 (11.2–96.5)	96.46 (37.0–181.4)	0.0001 *
Ferritin (ng/mL)—median (IQR)	655 (272–1194)	1128.3 (619.3–2085.3)	0.0001 *

IQR = interquartile range. CRP = C reactive protein; *—Mann–Whitney test; **—chi-square test.

**Table 3:** Biochemical parameters on the 14th day of hospitalization for the study groups.

Biochemical Parameters on the 14th Day of Hospitalization	Survivors (n = 143)	Deaths (n = 68)	Statistical Significance (*p*)
Leukocytes (×10^3^/mm^3^)—median (IQR)	8.38 (6.3–11.1)	9.29 (7.4–15.1)	0.0169 *
Neutrophiles (×10^3^/mm^3^)—median (IQR)	6.54 (4.4–9.4)	8.40 (6.5–13.4)	0.0004 *
Lymphocytes (×10^3^/mm^3^)—median (IQR)	1.08 (0.7–1.6)	0.58 (0.3–0.9)	<0.0001 *
Granulocytes/Lymphocytes ratio—median (IQR)	6.41 (2.9–10.4)	13.35 (8.5–25.9)	<0.0001 *
Procalcitonin (ng/mL):			
<0.5	115 (80.4%)	37 (54.4%)	0.0010 **
0.5–2	5 (3.5%)	6 (8.8%)	
2–10	1 (0.7%)	5 (7.4%)	
>10	0 (0.0%)	1 (1.5%)	
CRP (mg/L)—median (IQR)	11.54 (4–40)	41.28 (17.5–98)	<0.0001 *
Ferritin (ng/mL)—median (IQR)	647.4 (318.6–1123.5)	1197.1 (683.5–2397.8)	<0.0001 *

IQR = interquartile range. CRP = C reactive protein; *—Mann–Whitney test; **—chi-square test.

**Table 4:** Data related to ICU management of SARS-CoV2 infection for the two study groups.

	Survivors (n = 143)	Deaths (n = 68)	Statistical Significance (*p*)
Treatment with convalescent plasma—no. of patients (%)	3 (2.1%)	5 (7.4%)	0.1383 *
Treatment with immunomodulator (Tocilizumab)—no. of patients (%)	7 (4.9%)	4 (5.9%)	0.9762 *
Number of days in ICU—median (IQR)	12 (8–14)	13 (7–17.5)	0.2196 **

IQR = interquartile range; *—chi-square test with Yates’ correction; **—Mann–Whitney test.

**Table 5:** Relative risk factors for ICU death after SARS-CoV2 infection for studied parameters.

	Relative Risk	Confidence Interval 95%
Age	1.0749	1.0337—1.1178
The presence of obesity at ICU admission	6.0525	2.3121 to 15.8439
Procalcitonin > 10 ng/mL at ICU admission	4.23 × 10^6^	
G/L ratio on the 4th day of ICU	1.0922	1.0097 to 1.1814
Procalcitonin > 10 ng/mL at 4th day in ICU	4.02087	

G/L = granulocytes/lymphocytes ratio.

## Data Availability

The data presented in this study are available on request from the corresponding author.
